# The secret life of insect-associated microbes and how they shape insect–plant interactions

**DOI:** 10.1093/femsec/fiac083

**Published:** 2022-07-13

**Authors:** Silvia Coolen, Magda Rogowska-van der Molen, Cornelia U Welte

**Affiliations:** Department of Microbiology, Radboud Institute for Biological and Environmental Sciences (RIBES), Radboud University, Heyendaalseweg 135, 6525 AJ, Nijmegen, The Netherlands; Department of Microbiology, Radboud Institute for Biological and Environmental Sciences (RIBES), Radboud University, Heyendaalseweg 135, 6525 AJ, Nijmegen, The Netherlands; Department of Microbiology, Radboud Institute for Biological and Environmental Sciences (RIBES), Radboud University, Heyendaalseweg 135, 6525 AJ, Nijmegen, The Netherlands

**Keywords:** insect–microbe–plant interactions, insects, microbiota, pathogens, plant defenses, symbionts

## Abstract

Insects are associated with a plethora of different microbes of which we are only starting to understand their role in shaping insect–plant interactions. Besides directly benefitting from symbiotic microbial metabolism, insects obtain and transmit microbes within their environment, making them ideal vectors and potential beneficiaries of plant diseases and microbes that alter plant defenses. To prevent damage, plants elicit stress-specific defenses to ward off insects and their microbiota. However, both insects and microbes harbor a wealth of adaptations that allow them to circumvent effective plant defense activation. In the past decades, it has become apparent that the enormous diversity and metabolic potential of insect-associated microbes may play a far more important role in shaping insect–plant interactions than previously anticipated. The latter may have implications for the development of sustainable pest control strategies. Therefore, this review sheds light on the current knowledge on multitrophic insect–microbe–plant interactions in a rapidly expanding field of research.

## Teaser

A review on insect-associated microbes and their role in insect-plant interactions

## Introduction

Microbes have attained substantial attention in the past decades because of their importance in interactions with both animals and plants. Besides their ability to initiate serious infectious diseases with detrimental effects on their hosts, beneficial effects of microbes are also well-known. Microbes can support their animal hosts with digestion and the production of essential amino acids and vitamins. For plants, microbes have similar roles. Microbes in the soil decompose organic material and release nutrients, they can promote plant growth by facilitating efficient nutrient uptake and support the plant’s defensive system by priming plant responsiveness to potential threats (Van Oosten et al. 2008, Oldroyd et al. 2011, Pieterse et al. 2012, Trivedi et al. 2020).

In the animal kingdom, under the phylum arthropoda, there are an estimated number of 5.5 million insect species of which currently approximately one million species have been named and compiled in the catalog of life. About half of these insects are plant feeding (Schoonhoven et al. 2005, Bernays 2009, Zhang 2011, Stork 2018). In addition, approximately 6000 mite species affiliated with arachnids form an important class of plant feeding insects. Important agricultural, horticultural, and forestry pests are mainly found in the insect orders of plant chewing Orthoptera (e.g. grasshoppers), Hymenoptera (e.g. sawflies), Coleoptera (e.g. beetles), Lepidoptera and Diptera (e.g. caterpillars and flies), insects of the piercing and sucking insect orders of Hemiptera and Thysanoptera (e.g. aphids and thrips), and the class of joint-legged invertebrates (Arachnida) with the orders Tetranychidae and Eriophyoidae (e.g. spider mites and gall mites (Malais and Ravensberg 2004, Bernays 2009, Stork 2018).

Approximately 18%–20% of crop losses are estimated to be caused by direct damage by insects and roughly 30%–40% of the yield reduction of crops is estimated to be due to the combination of insects and indirect effects of microbial transmission by insects (Weintraub and Beanland 2006, O’Hara et al. 2008, Sharma et al. 2017). Insect host range is largely depending on adaptations that developed during coevolution with plants, resulting in insects that are restricted to particular plant families or species (i.e. specialists; oligophage or monophage), whereas others have a very broad host plant range (i.e. generalists; polyphage (Ali and Agrawal 2012). Major food crops that are affected by insects are found within the top 10 crops with highest agricultural gross production value (FAO 2016) including rice, maize, wheat, soybean, tomato, potato, sugar cane, vegetables (e.g. bamboo shoots, beets, celery, parsley, and so on), grapes, and apples. Floriculture and forestry are also of major economic importance and deal with various insects. Floriculture often involves greenhouse cultivation that are ideal environments for thrips and aphids, whereas forestry deals with large chewing insects such as grasshoppers and beetles (Malais and Ravensberg 2004, CABI 2020).

To many plant feeding insects, microbes are essential. Without their symbiotic microbiota insect health and survival is severely impacted or life is even impossible (Douglas 2017, Singh et al. 2019). In addition, microbes associated with insects have shown to shape the interactions between insects and their host plants (Frago et al. 2012). Since 80% of the approximately 900 known plant viruses are transmitted by insect vectors, this review will only briefly touch upon viral transmission and its effect on insect–plant interactions (Hohn 2007, Roossinck 2012). To obtain a better understanding of the multitrophic interactions between insects and their host plants, this review focuses on the role of insect-associated microbes in shaping insect–plant interactions.

### Insect microbiota

Insects are associated with a plethora of microorganisms including transient microorganisms, which can be pathogenic to either the insect or the host plant (phytopathogens), and symbionts that can have a relationship with their host in which both benefit (mutualism), none benefit (commensalism), or one benefits while the other is harmed (parasitism; Perlmutter and Bordenstein 2020). These microbes include bacteria, archaea, fungi, protists, and viruses, and originate from insects or are transferred by insects between visited host plants (Frago et al. 2012, Perilla-Henao and Casteel 2016).

### Transient microbiota, plant beneficial microbes, and pathogens

Transient insect microbiota is temporarily associated with their host and obtained via, and reflective of, the environment, including soil and plant-associated microbes, plant symbionts (e.g endophytes), plant beneficial and growth promoting microbes, and pathogens (Muratore et al. 2020). Plant piercing and sucking insects such as mirids and leafhoppers (Hemiptera) were recently described in transmitting plant beneficial endophytes that promote plant growth and with that also indirectly support their host insect with sufficient food material (Lopez-Fernandez et al. 2017, Galambos et al. 2021). Likewise, transmission of plant pathogens can occur. An example is the transmission of the notorious hemibiotrophic plant pathogen *Pseudomonas syringae* of which its pathovars can infect most important crop species worldwide (Xin et al. 2018). Transmission of this bacterium by the citrus flatid planthopper (*Metcalfa pruinosa*) causes bacterial canker in kiwifruit plants (Donati et al. 2017). However, *P. syringae* was also shown to repress plant defenses that are harmful to insects, making them ideal partners for insects (Groen et al. 2016). In contrast, some leaf associated *P. syringae* strains are pathogenic to insects, including aphids, changing the plant–insect interaction outcome (Smee et al. 2021).

### Horizontal and vertical transfer of insect symbionts

For many insects symbiotic microbes are essential and life without them is impossible or severely affected. Insect microbial symbionts play important roles in digestion, nutrition, and protection of their host against pathogens (Dillon and Charnley 1995, Chevrette et al. 2019, Ankrah et al. 2020). Especially insects feeding from plant xylem and phloem sap, i.e. deficient in essential nutrients, rely on microbial supplementation of essential amino acids (Douglas 2006). However, some insects seem to be less affected by removal of their microbiota, these include caterpillars (Lepidoptera), grasshoppers (Orthoptera), thrips (Thysanoptera), and spider mites (Tetranychidae; Charnley et al. 1985, Whitaker et al. 2016, Hammer et al. 2017, 2019, Phalnikar et al. 2019). The presence of microorganisms in the aforementioned insects may still give rise to beneficial properties (Dillon and Charnley 1995, Idowu et al. 2009, Chevrette et al. 2019). Depending on whether insects rely on symbionts for essential or beneficial services (i.e. obligate versus facultative symbionts), microbes can either be obtained via horizontal or vertical transmission. Obligate symbionts are transferred vertically via the mother to offspring before or during birth, via egg surfaces or specific behavior that allows for the transfer of essential microbes. Facultative symbionts, that facilitate favorable nonessential tasks for their host, are usually obtained from the environment through feeding or contact leading to horizontal transmission (Kikuchi et al. 2011a, Caspi-Fluger et al. 2012, Hannula et al. 2019). Mixed modes of transmission also exist in which symbionts can be obtained both vertically and horizontally, called pseudo-vertical transmission (Bright and Bulgheresi 2010). Facultative symbionts fulfil highly diverse roles for their host including high-temperature tolerance, sex determination, and body coloration (Montllor et al. 2002, Dillon and Dillon 2004, Werren et al. 2008, Tsuchida et al. 2010). For instance, red-colored pea aphids infected with a *Rickettsiella* facultative endosymbiont turned green due to the production of blue–green polycyclic quinones (Tsuchida et al. 2010).

### Diversity of insect symbionts

Although research on insect microbiota is a rapidly expanding field, we are only starting to understand the diversity and complexity of the microbial communities associated with insects. Insect symbionts include microbes such as *Burkholderia, Buchnera, Wolbachia, Pantoea, Sodalis, Carsonella, Portiera, Pseudomonas, Phytoplasma, Spiroplasma, Rickettsia, Arsenophonus, Cardinium, Serratia, Stammera, Arsenophonus, Blattabacterium, Blochmannia, Rhodococcus, Wigglesworthia, Nasuia, Morganella, Riesia, Coxiella, Asaia, Baumannia, Hamiltonella, Moranella, Nardonella, Nasuia, Sulcia, Zinderia, Tremblaya, Uzinura, Hodgkinia, Regiella*, and many other (uncultured) bacteria (Shikano et al. 2017, Perlmutter and Bordenstein 2020). Some of these insect symbionts are also phytopathogenic (Box 1). Besides bacteria, insects were shown to be colonized by diverse yeasts and molds or even archaeal methanogens (Tokura et al. 2000, Idowu et al. 2009, Gomez-Polo et al. 2017, Kobialka et al. 2018, Matsuura et al. 2018). However, studies on nonbacterial communities of insect inhabitants remains poorly explored and will not be covered in this review article.

Box 1: **Examples of phytopathogenic insect symbionts**

- *Phytoplasma* are a very diverse group of Gram-positive, pleomorphic-shaped phytopathogenic bacteria that colonize both insects and plants intracellularly (Sugio et al. 2011). Because they colonize host cells, they benefit from cellular processes of the host and do not require complex genomes. *Phytoplasma* have the smallest genomes of all described phytopathogenic bacteria, averaging ∼0.7 Mb with a low G+C content (Kube et al. 2012). They have a wide host range, infecting more than 800 different plant species and causing more than 1000 plant diseases (Mitchell 2004, Weintraub and Beanland 2006, Hogenhout et al. 2008). Hemipteran insects are most successful in transmitting phytoplasma’s.
- *Spiroplasma* are Gram-positive helical-shaped intracellular bacteria that are distantly related to *Phytoplasma* and of which only some are phytopathogens (Ammar et al. 2004, Sugio et al. 2011, Perilla-Henao and Casteel 2016). *Spiroplasma kunkelii* causes corn stunting disease, i.e. transmitted by leafhoppers and in severe cases leads to the complete loss of corn seed production (Özbek et al. 2003, CABI 2020). *Spiroplasma citri* causes citrus stubborn disease and is transmitted by leafhoppers to other plant species including carrot and periwinkle, causing leaf discoloration (Mello et al. 2009).
- *Rickettsia*-like organisms (RLOs) are intracellular Gram-negative bacteria that are present in insects and fulfill roles in primary nutrition. They can manipulate insect reproduction and are transmitted to plants where they are pathogenic. Insects transmitting RLOs are white flies, cicadas, leafhoppers, and psyllids (Caspi-Fluger et al. 2012, Constable and Bertaccini 2017). In plants, RLOs cause strawberry lethal yellows, grape vine yellows, strawberry green petal, Papaya bunchy top disease, and a couple of other diseases (Davis et al. 1996, Streten et al. 2005).
- *Pantoea* *ananatis*, *P. agglomerans*, and *P. stewartii* are Gram-negative plant epiphytic and insect (endo-)symbiotic bacteria that are transmitted by insects including thrips, flea beetles, shield bugs, and false potato beetle (*Leptinotarsa juncta*), and are present in different life stages of their insect vector (Gitaitis et al. 2003, Coutinho and Venter 2009, Ammar et al. 2014, Dutta et al. 2016). *Pantoea* have diverse roles in their host insects, including nitrogen fixation, nutrient supplementation, digestion, and detoxification (MacCollom et al. 2009, Walterson and Stavrinides 2015). *Pantoea* spp. are known to be transmitted via insect frass and cause galling, wilting, soft rot, and necrosis in a variety of agricultural crop plants (Walterson and Stavrinides 2015). *Pantoea agglomerans* can be transmitted by *Nezara viridula* shield bugs and causes boll rot of cotton (Medrano et al. 2007). The latter is reported to cause 10%–15% of cotton yield losses in the USA (Hollis 2001). Both *P. agglomerans and P. ananatis* transmitted by onion thrips (*Thrips tabaci*) can cause center rot of sweet onion (*Allium cepa*; Dutta et al. 2014). *Pantoeastewartia* can be transmitted by maize flea beetles (*Chaetocnema pulicaria* and *C. denticulate*) and causes Stewart’s bacterial wilt and leaf blight in maize (Correa et al. 2012).
- *Liberibacter* spp. are Gram‐negative insect symbionts that live as phloem‐limited obligate microorganism in plants and are associated with several plant diseases. Citrus huanglongbing (yellow shoot) or citrus greening disease is associated with three different *Ca*. Liberibacter species, namely *Ca*. Liberibacter asiaticus, *Ca*. L. africanus, and *Ca*. L. americanus. *Ca*. L. asiaticus is the most severe pathogen, spread by Asian citrus psyllid *Diaphorina citri* and causes devastating epidemics in several countries. *Ca*. *L. africanus* occurs in Africa, where it is spread by the African citrus psyllid *Trioza erytreae*. *Ca*. L. solanacearum is associated with diseases, e.g. causing Zebra chip symptoms in several solanaceous plants (e.g. potato, carrot, and celery), and transmitted by potato psyllid *Bactericera cockerelli*, and the psyllids *Trioza apicalis* and *Bactericera trigonica* (Haapalainen 2014).
- *Xylella* *fastidiosa* is a plant xylem colonizer that colonizes the surface of insect’s foregut and is transmitted by a diverse set of xylem-feeding hemipterans such as leafhoppers and spittlebugs, where it colonizes the narrow canal of the chitinous mouthparts (Almeida et al. 2005, Perilla-Henao and Casteel 2016). When coming into contact with plant pectin, vector transmission of *X. fastidiosa* is induced (Killiny and Almeida 2009). *Xylella* produces enzymes involved in the degradation of pectin, glucan, and cellulose (Roper et al. 2007). *Xylella fastidiosa* has a very wide host range covering over 60 plant families, causing Pierce’s disease in grapes, variegated chlorosis in citrus plants, leaf scorch in olive and almond trees, and several other diseases in other plant species (EFSA Panel on Plant Health 2015).

### Insect organs that harbor symbionts

Insect symbionts can be located on both the exterior and interior of insects. Leafcutter ants, that strip down tree leaves to cultivate a *Leucoagaricus* fungus as their food supply, carry symbiotic *Pseudonocardia* bacteria on their exterior that protects *Leucoagaricus* from a devastating parasitic fungus named *Escovopsis* (Heine et al. 2018). However, the majority of insect symbionts are more closely associated to their host insect and live inside the gut, haemolymph, malpighian tubules, fat body, specialized symbiont organs (i.e. bacteriomes and bacteriocytes in aphids), and reproductive organs (Tada et al. 2011, Perlmutter and Bordenstein 2020). Due to their reduced genomes, obligate insect symbionts rely on host metabolism and, therefore, evolved intimate relationships with their host and can often be found as endosymbionts within the insects or even within cells that provide protection against the insect’s immune system (Moran et al. 2009, McCutcheon and Moran 2010, Wilson and Duncan 2015, Chung et al. 2018).

### Symbiont and cosymbiont adaptation

Many endosymbionts rely on the metabolism of their host and other symbionts for survival and reproduction, and together with their high mutation rate this causes gene losses that lead to small endosymbiont genomes (Moran et al. 2009, McCutcheon and Moran 2010). Eventually, these symbionts may accidentally lose genes that are essential to their host resulting in its replacement by other symbionts. An example of such integrated metabolic processes is the *Sulcia* obligate symbionts of xylem-feeding insects such as spittlebugs, sharpshooters, and cicadas (Hemiptera). *Sulcia* provides its host with 8 out of 10 essential amino acids (arginine, isoleucine, leucine, lysine, phenylalanine, threonine, tryptophane, and valine; Ankrah et al. 2020). Co-obligate symbionts *Zinderia*, *Sodalis*, *Baumannia*, and *Hogdkinia* cover the other essential amino acids including methionine and histidine that are not produced by the primary obligate symbiont. In another study, *Sulcia*-CARI of sharpshooters was shown to have lost its tryptophane biosynthetic pathway that was successfully compensated with tryptophane production by co-obligate symbiont *Zinderia*, although the latter only has a very small genome of 208 kb (Moran et al. 2009, McCutcheon and Moran 2010). Another example is *Buchnera*, the endosymbiont of aphids that exhibits ongoing gene loss (Lamelas et al. 2011). The *Buchnera aphidicola* symbiont of aphid *Cinara cedri* has lost its ability to produce tryptophane and riboflavin, whereas coexisting endosymbiont *Serratia* was found to be able to produce tryptophane, covering essential metabolism for both endosymbionts (Lamelas et al. 2011). Moreover, extreme genome reductions of obligate symbionts may result in symbionts having their own endosymbionts, such as the mealybug *Tremblaya* symbiont’s endosymbiont *Moranella* (Husnik et al. 2013). In other cases, bacterial obligate symbionts may be replaced by eukaryotes as in the case of cicadas, where the lack of bacterial *Hodgkinia* symbionts resulted in replacement with a yeast-like fungal associate, indicating the importance of the microbial community beyond bacteria (Gomez-Polo et al. 2017). Therefore, microbial insect symbiosis seems to be a more dynamic state of co-operation than a static relationship.

### Microbial transmission from insects to plants

Since microbes are essentially everywhere, transmission is inevitable. During insect feeding, microbes are obtained from colonized tissues and subsequently transmitted via secreted saliva and oral secretions in the form of regurgitant that originates from the anterior part of the insect’s gut system, or via frass (Mitchell and Hanks 2009, Chung et al. 2013, Felton et al. 2014). Plant-sap feeding insects are most successful in transmitting microbes to plants due to their nondestructive feeding strategy that allows microbes to safely pass plant physical and chemical barriers. However, leaf chewing insects also transmit microbes into open wounds, exposing microbes to toxic chemical defenses of damaged plants. Depending on the insect’s plant host range, specialist insects are likely to only transmit microbes to a very limited group of plant species, whereas generalists have the potential to inoculate a wider range of plant species and with that also potentially indirectly transmit microbes to specialist insects (Kingdom and Hogenhout 2007, Mello et al. 2009). The latter could maybe lead to microbial adaptations of nonpest insects that could allow them to feed on other plant species (Hosokawa et al. 2007).

### Insect microbiota and pest status

It has been shown that exchange of gut microbiota of pest insects to nonpest insects can lead to an obtained pest-status. When soybean pest shield bug *Megacopta punctatissima* and nonpest *M. cribraria* egg-transmitted symbionts were swapped, this led to a reversal of insect performance on soybean (Hosokawa et al. 2007). Also for pea aphids (*Acyrthosiphon pisum*) their microbiota was shown to be essential for enabling efficient reproduction on specific plants (Tsuchida et al. 2004). While leaving their obligate symbiont *Buchnera* unharmed, the microbiota of genetically identical animals was eliminated using antibiotics, selectively excluding the offspring’s microbiota. Offspring of antibiotic-treated insects reproduced equally well on vetch plants (*Vicia sativa*), whereas they lost almost 50% fecundity on white clover (*Trifolium repens*) in comparison with control offspring, again showing the importance of the insect microbiota for host–plant interactions (Tsuchida et al. 2004). Phytoplasmas that are transmitted via insect vectors are also known to alter insect host range via, yet, unknown mechanisms. Aster yellows phytoplasmas (AYPs), on the one hand, increase the fecundity of their vector, e.g. the generalist leafhopper *Macrosteles quadrilineatus* that transmits AYPs to diverse plants. On the other hand, the monophage leafhopper *Dalbulus maidis* survives longer on AYP-infected nonhost plants, indicating that spreading of AYPs by generalist leafhoppers has implications for the host range of specialist leafhoppers (Kingdom and Hogenhout 2007). A mechanism via which insect behavior and pest status is changed is indicated by the “vector manipulation” hypothesis (Ingwell et al. 2012). This hypothesis states that the insect’s host plant selection is manipulated by microorganisms in order to support their spreading. Corn-specialist leafhopper *D. maidis* was shown to be attracted to plants infected with phytoplasma and subsequently prefer healthy plants, increasing the spread of infection, whereas oviposition on infected plants was drastically reduced (Ramos et al. 2020). The underlying mechanisms of such interactions are yet to be revealed. Since polyphagous insects have the potential to visit a wide range of plant species, transmission of microbes including symbionts through plant surfaces may facilitate the sharing of symbionts among different insect species with unknown consequences. Furthermore, transmitted microbes may alter plant physiology and defense state potentially enabling insects to feed from defense compromised plants that were otherwise inaccessible.

### Shaping insect–microbe–plant interactions

**Figure 1.**
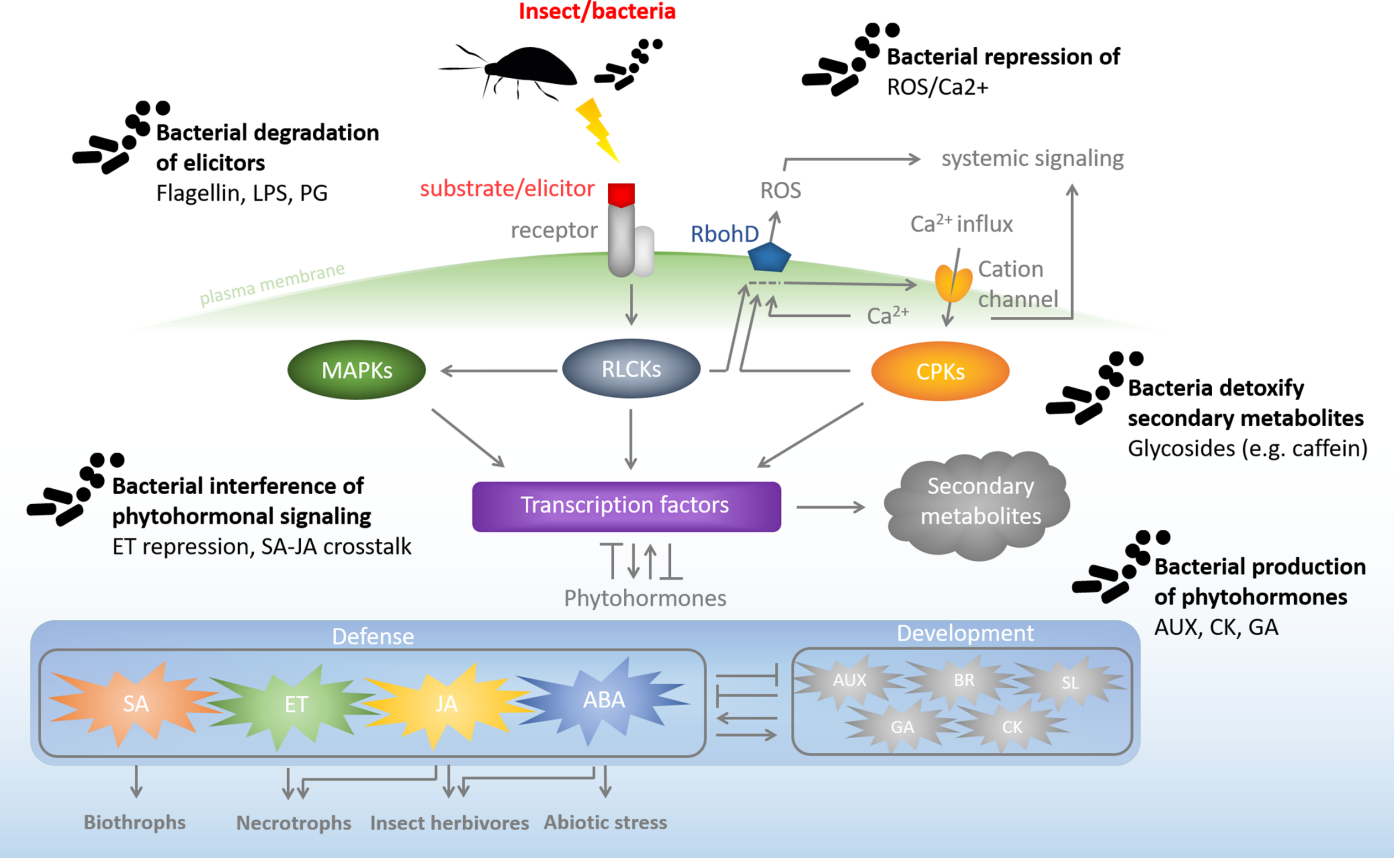
**Overview of insect-associated microbe interference with plant defense signaling**. Plant stress perception leads to the activation of receptor-like cytoplasmic kinases (RLCKs), mitogen activated kinases (MAPKs), and Ca^2+^ influx, which in turn results in the activation of calcium-dependent protein kinases (CPKs). Ca^2+^, RLCKs, and CPKs are involved in the activation of RbohD, which produces extracellular reactive oxygen species (ROS) that together with Ca^2+^ acts as second messenger in systemic signaling throughout the plant. Activation of CPKs, RLCKs, and MAPKs leads to downstream stress signaling, involving the activation of transcription factors that regulate the production of phytohormones and secondary metabolites. Crosstalk between (phytohormonal) signaling pathways is further explained in the main text. ABA, abscisic acid; AUX, auxin; BR, brassinosteroids; CK, cytokinins; ET, ethylene; GA, gibberellin; JA, jasmonic acid; LPS, lipopolysaccharides; PG, peptidoglycan; SA, salicylic acid; and SL, strigolactones.

When it comes to preventing insects and microbes from exploiting plant resources, plants are not helpless. Plants have evolved sophisticated defense systems including constitutive physical and chemical barriers, stress specific detection systems, downstream cross-communicating phytohormonal defense signaling pathways, enhanced defense mechanisms that enable fast responses, and the production of secondary metabolites to ward off invading threats and attract insect parasitoids (Fig. 1; Pieterse et al. 2012, 2014, Stam et al. 2014).

### Breaching and benefitting from plant physical barriers

As a first line of defense, plants have constitutive physical and chemical barriers (e.g. reinforced cell walls, waxy cuticles, trichomes and preformed metabolites, and antifeeding compounds) that are meant to ward off invading insects and microbes (Schoonhoven et al. 2005). Microbes can help insects to overcome these first lines of plant defense and even benefit from it since these barriers form a rich carbon source of organic polymers and complex polysaccharides, e.g. pectin, lignin, and cellulose. During feeding, chewing insects produce oral secretions that consist of saliva and regurgitant-containing enzymes that help digest plant material and support nutrient uptake (Bonaventure et al. 2011). Insects that lack essential digestive enzymes such as the tortoise beetle *Cassida rubiginosa* can make use of symbionts such as *Ca*. Stammera capleta that supports pectin degradation with the help of two secreted pectinolytic enzymes. For the Tortoise beetle, these microbial enzymes are essential for beetle survival (Salem et al. 2017). In larvae of root feeding white grub beetles, *Lepidiota mansueta*, cellulose degrading *Citrobacter* (*Enterobacteriaceae*) bacteria were found likely supporting their host with breakdown of cellulose (Handique et al. 2017). Also, in the phytophagous Forest Cockchafer (*Melolontha hippocastani*, *Coleoptera*) Enterobacteriaceae were found to be most active in cellulose degradation, shown by high ^13^C isotope-labeled carbon incorporation into bacterial DNA after insect feeding from ^13^C-cellulose (Alonso-Pernas et al. 2017). Cellulose and lignin degradation in *Reticulitermes flavipes* termites was found to rely on both symbiotic protists and their host, although it remains largely uncharacterized to what extend the symbionts are responsible for lignin digestion because of the obligate symbiotic nature of their relationship (Raychoudhury et al. 2013). Plant preformed chemical barriers consist of constitutively produced metabolites (i.e. toxins) such as breakdown products of (e.g. sulfur and nitrogen containing-) glycosides (e.g. isothiocyanates) that can also be used as nitrogen and carbon sources (see “Microbial detoxification of plant defensive compounds;” Kos et al. 2012, Yang et al. 2018).

### Preventing recognition by the plant

As a postinvasive line of protection, plants evolved an innate immune system by which they recognize nonself-molecules and signals from stressed or injured cells and respond by activating a stress specific counter response (Fig. 1; Jones and Dangl 2006, Howe and Jander 2008). When insects start feeding on host plants, they produce digestive enzymes like glucose oxidase, β-glucosidase, and pectinase, and release insect-associated molecules such as lipids, fatty acids, and fatty acid conjugates into the plant (Yoshinaga et al. 2007, 2008, Van Doorn et al. 2010, Bonaventure et al. 2011). These molecules are known as herbivore associated elicitors (HAEs) or herbivorous-insect associated molecular patterns (HAMPs) that can be recognized by the plant’s pattern recognition receptors (PRRs) and elicit a plant defense response. Like insects, microbes can also be recognized by plants via similar molecules, called microbe or pathogen associated molecular patterns (MAMPs or PAMPs). These molecules include fungal chitin, bacterial elongation factor thermo-unstable (EF-TU), lipopolysaccharide (LPS), peptidoglycan (PGN), and flagellin (FLG; Zipfel et al. 2006, Chinchilla et al. 2007, Yamaguchi et al. 2010, Couto and Zipfel 2016). In addition, insect feeding or microbial invasion can result in the release of plant-derived elicitors, called damage-associated molecular patterns (DAMPs, e.g. peptides and oligogalacturonides), that are part of the plant’s wound response (Schmelz et al. 2006, Bonaventure et al. 2011, Li et al. 2020). Recognition of elicitors by PRRs initiates a defense response along with stress-specific downstream signaling. Plants distinguish between different kinds of stress by making use of stress-specific PRRs eliciting specific responses, leading to pattern-triggered immunity. However, this response can be breached with effector molecules of invading insects and microbes, targeting the plant’s defensive signaling, leading to effector triggered immunity when plants intercept these manipulative molecules with intracellular resistance proteins (Jones and Dangl 2006).

When insects and their associated microbes are recognized by host plants, effective pattern-triggered immunity defenses are activated. However, microorganisms have developed mechanisms to prevent recognition by the plant to ensure an undisturbed invasion into the host plant. For instance, the notorious plant pathogen *P. syringae*, i.e. vectored by insects, can prevent recognition of their flagellin molecules by the plant via the secretion of flagellin-degrading alkaline protease A (AprA; Bardoel et al. 2011, Pel et al. 2014). In both plants and human cell cultures, this mechanism was shown to be effective in preventing bacterial recognition, making bacterial invaders invisible for plants. For another insect-vectored plant pathogen, *X. fastidiosa*, it was shown that the production of an LPS O-antigen delays LPS recognition in plants and with that also an effective pattern triggered immunity response against the pathogen (Rapicavoli et al. 2018). Besides preventing their own recognition, insect-associated microbes could potentially also prevent their host insect from being recognized by the host plant. For *Wolbachia* symbionts it was shown that their small noncoding RNAs could affect insect host genes, potentially indirectly influencing HAMPs that are recognized by plants (Barr et al. 2010, Shikano et al. 2017).

### Interference of early plant defense signaling

Directly after recognition of a potential threat, plant receptor binding leads to local phosphorylation of receptor kinases, release of glutamate, rapid calcium (Ca^2+^) influx, and phosphorylation of downstream receptor-like cytoplasmic kinases (RLCKs) and calcium-dependent protein kinases (CPKs) that recruit and phosphorylate respiratory burst oxidase homologue D (Fig. 1; RbohD; Mersmann et al. 2010, Ranf et al. 2011, Dubiella et al. 2013, Liu et al. 2013, Kadota et al. 2014, Kim et al. 2022). Activation of RbohD results in the production of extracellular reactive oxygen species (ROS) that depolarizes plant cells within minutes after recognition of an elicitor. Both Ca^2+^ and ROS act as second messengers activating plant stress signaling throughout the whole plant with an astonishing speed of up to 2.4 cm min^–1^ and 8.4 cm min^–1^, respectively (Chinchilla et al. 2007, Jeworutzki et al. 2010, Mittler et al. 2011, Suzuki et al. 2013, Choi et al. 2014, Couto and Zipfel 2016). The release of glutamate was shown to be a key player in facilitating Ca^2+^ long distance signaling via the activation of glutamate receptor-like cation-permeable ion channels (Toyota et al. 2018). Furthermore, Ca^2+^ signaling has an elicitor-specific signature and amplitude, indicating that this general defense signal might carry stress-specific information (Ranf et al. 2011).

For piercing and sucking insects, blocking of Ca^2+^ signaling is essential in order to prevent clogging of plant sieve elements that are required for providing phloem sap (Will et al. 2007, 2013). Bacteria transmitted by insects are also known to manipulate plant defense signaling by interfering with the calcium signaling. Phytopathogenic *P. syringae* bacteria, that are transmitted by many insects including leaf mining fly larvae, target the plant’s Ca^2+^ sensor calmodulin, thereby affecting the production or ROS and rendering the plant more susceptible to the pathogen (Groen et al. 2016, Guo et al. 2016). ROS are well-known for their antimicrobial nature, therefore, microbes try to limit their production and with that simultaneously support insects that feed on the same plant. *Pseudomonas syringae* was shown to enhance insect herbivory by leaf mining fly larvae (*Scaptomyza flava*) in *Arabidopsis*, by suppressing the ROS-burst after recognition (Groen et al. 2016). *S. flava* larvae prefer to feed and develop faster on *P. syringae* infected leaves, confirming their beneficial effect on insects. Besides rapid second messenger (i.e. Ca^2+^ and ROS)-induced signaling, plant receptor activation leads to downstream receptor like kinase (RLK) and mitogen activated kinase (MAPK) signaling that activates transcription factors (TFs) involved in stress signaling regulation, cross-communication between different stress signaling pathways, and amplification of phytohormone-driven and plant stress responsive pathways (Asai et al. 2002, Du et al. 2009, Mittler et al. 2011, Gao et al. 2013, Suzuki et al. 2013, Couto and Zipfel 2016). These downstream signaling pathways are also targets of bacteria.

### Phytohormones

Plant hormones play a key role in regulation and amplification of plant defenses (Boutrot et al. 2010, Mersmann et al. 2010, Qiu et al. 2014). Depending on the nature of the stress, plants make use of phytohormone-driven signaling pathways, including the production and accumulation of abscisic acid, salicylic acid, jasmonic acid, and ethylene (Fig. 1; Anderson et al. 2004, Robert-Seilaniantz et al. 2011, Pieterse et al. 2012). Plant responses to different biotic and abiotic stresses requires the action of one or several phytohormones subsequently or simultaneously (Glazebrook 2005, Qin et al. 2011). Plant responses to biotrophic pathogens that feed from living cells generally induce salicylic acid defenses. In case of necrotrophic pathogens that feed from dead plant tissues, jasmonic acid and ethylene defenses are activated. Hemi-biotrophic pathogens (such as *P. syringae*) induce plant salicylic acid defenses followed by jasmonic acid in their necrotrophic stage. In response to chewing herbivores jasmonic acid and abscisic acid are induced, whereas with abiotic stress abscisic acid is the responsive hormone. Jasmonic acid signaling results in two distinct antagonizing signaling branches modulated by ethylene and abscisic acid (Anderson et al. 2004, De Vleesschauwer et al. 2010, Pieterse et al. 2012). Besides phytohormone stress-driven plant signaling pathways, plants make use of development and growth related phytohormones auxin, gibberellin, cytokinins, brassinosteroids, and strigolactones that are closely interacting and cross-communicate with defense hormones (Peleg and Blumwald 2011, Pieterse et al. 2012, Waters et al. 2017).

Bacteria are known to produce phytohormones and their mimics and have a plethora of adaptations that allow them to influence respective plant signaling pathways to steer their interactions. Plant beneficial endophytes, that are transmitted by piercing and sucking insects, are known to promote plant growth, via enhanced nutrient acquisition (e.g. nitrogen fixation) or modulation of plant hormones such as auxin, cytokinin, and gibberellin (Lugtenberg and Kamilova 2009). Bacteria are well-known for their ability to produce the main plant auxin, namely indole-3-acetic acid, including bacteria such as *Pseudomonas, Rhizobium, Azospirillum, Enterobacter, Azotobacter, Klebsiella, Alcaligenes, Pantoea, Acetobacter, Herbaspirillum, Burkholderia, Bacillus, Rhodococcus, and Streptomyces* (Ali et al. 2017). The latter is no surprise, since it was recently discovered that plant indole-3-acetic acid production originates from bacterial horizontal gene transfer, explaining their importance for both organisms and in interactions between them (Bowman et al. 2021). Both insects and microbes make use of plant auxin signaling to promote plant growth to ensure sufficient food recourses for their development (Machado et al. 2013, 2016, Coolen et al. 2016, Davila Olivas et al. 2016). Besides promoting plant growth via indole-3-acetic acid, plant endophytes are known to promote plant growth by lowering plant stress hormone ethylene via the production of the enzyme 1-aminocyclopropane-1-carboxylase deaminase (Rashid et al. 2012). Also, plant beneficial bacterium *Klebsiella oxytoca* promotes plant growth via ethylene repression (Glick et al. 2007, Kifle and Laing 2016), which most probably affects plant defenses against pathogens and insects involving ethylene signaling. Insect-transmitted phytopathogen *P. syringae* also suppresses ethylene signaling, preventing ethylene-induced stomatal closure with its HopM1 effector to ensure easy passage into the plant’s apoplast (Lozano-Durán et al. 2014). In addition, *P. syringae* represses expression both PAMP (flagellin and EF-Tu) and DAMP-induced plant stress signaling with the help of its AvrPto effector molecule (Gravino et al. 2017). Since DAMP-responses are also present in plant defenses against chewing insects, *P. syringae* AvrPto may be beneficial to insects.

Some bacteria are also known to produce cytokinins that directly influence plant physiology. An example is the insect symbiont *Wolbachia* of leaf miner caterpillars (*Phyllonorycter blancardella*, Lepidoptera) that produces cytokinins that cause a “green island” phenotype of photosynthetically active green patches and, thereby increase the viability of its insect host due to an increase in plant chlorophyll content (Kaiser et al. 2010). *Phytoplasma* infection was also shown to be correlated with plant hormonal imbalance that extends to uninfected tissues, leading to a favorable nutritional status of the plant for insects (Pradit et al. 2019). However, cytokinin production may also turn commensal plant bacteria into potential phytopathogens. The plant commensal and insect symbiont *P. agglomerans* has apparently acquired a plasmid containing cytokinin biosynthesis genes that turn the bacterium into a gall-forming plant pathogen (Barash and Manulis-Sasson 2007, Medrano et al. 2007). Furthermore, galling mite *Fragariocoptes setiger* (Eriophyoidea) was shown to harbor other microbiota with the potential to form plant galls, including *Agrobacterium*, *Rhodococcus*, *Pseudomonas*, and *Erwinia* (Klimov et al. 2022). Furthermore, the plant pathogen *Xanthomonas oryzae* pv. oryzicola produces bioactive gibberellin that reduce plant jasmonic acid defenses, thereby indirectly benefitting insect feeding on the affected plant. The gibberillin biosynthesis pathway in bacteria was shown to be identical to that of plants, again indicating the potential of cross-kingdom communication and potential horizontal gene transfer (Nagel et al. 2017).

### Interference of stress phytohormones and cross-communication

**Figure 2.**
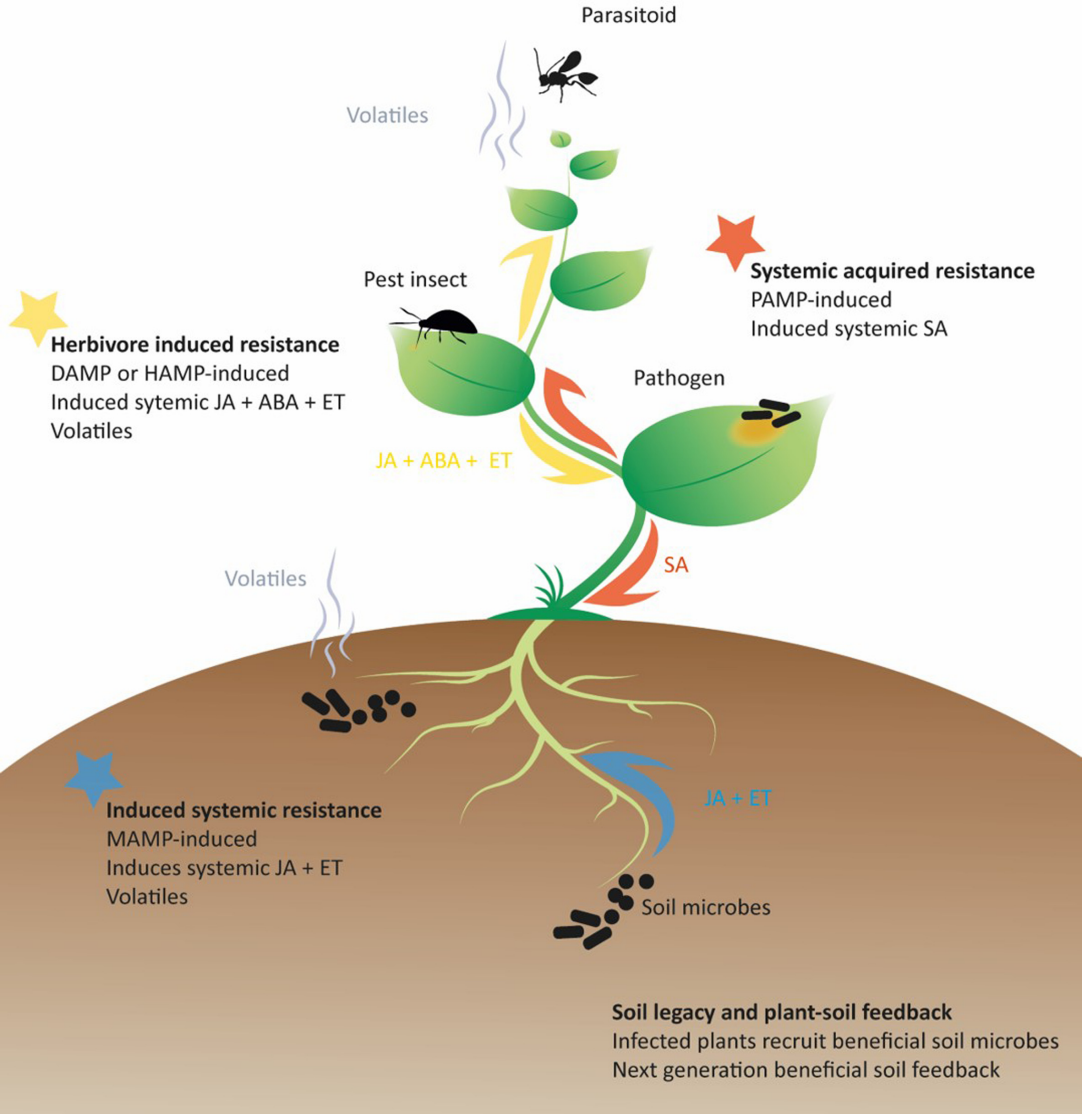
**Schematic overview of major induced systemic plant defense pathways**. Herbivorous insects induce damage or herbivore (DAMP/HAMP)-induced resistance (in yellow) in plants that gives rises to defense priming for jasmonic acid (JA), abscisic acid (ABA), and ethylene (ET). Herbivore-triggered resistance also involves the production of herbivore-induced plant volatile emission that attracts insect parasitoids that parasitize insects. Pathogenic microbes, that are often transmitted by insects, can trigger plant PAMP-induced systemic acquired resistance (SAR, in red) that primes plant defenses for salicylic acid (SA). Plant beneficial soil-microbes, potentially transmitted by insects, can give rise to induced systemic resistance (ISR) that primes plants for JA and ethylene defenses and subsequent release of volatiles. Infected or infested plants also recruit beneficial microbes that protect next generations of plants against infestation via a microbial soil legacy through plant–soil feedback.

To cope with a multitude of different stresses, plants rely on a well-balanced and cross-communicating signaling system (Fig. 2). Stress signaling is often found to negatively regulate plant growth and development, suggesting that plant stress signaling is prioritized over growth and development. In contrast to that, plant growth-related signaling negatively regulates stress signaling, and both insects and microbes make use of this antagonism (Kazan and Manners 2009, Hentrich et al. 2013). In crosstalk between plant phytohormonal pathways, antagonism and synergism have been extensively described and are highly complex. Within the set of phytohormones known for their cross-communication abilities, salicylic acid and jasmonic acid are the most extensively studied (Laurie-Berry et al. 2006, Mur et al. 2006, Robert-Seilaniantz et al. 2011, Caarls et al. 2015). Salicylic acid-mediated suppression of jasmonic acid signaling is responsible for increased plant susceptibility to many insects and is, therefore, a core target in insect–plant interactions.

The saliva and oral secretions of insects that are transferred to plants during feeding are sufficient to repress undesired jasmonic acid defenses (Verhage et al. 2011). Furthermore, oral secretions can contain microbes that are transferred to the plant during feeding and induce plant defenses that interfere with normal plant responses to invading insects. For both the Colorado potato beetle (*Leptinotarsa decemlineata*) and its closely related false potato beetle (*L. juncta*) it was demonstrated that *Stenotrophomonas, Pseudomonas, Enterobacter*, and *Pantoea* bacteria within oral secretions induce plant salicylic acid defenses, thereby repressing jasmonic acid-defenses, leading to optimal growth of the beetles (Chung et al. 2013, Wang et al. 2016, Sorokan et al. 2019). Also phloem-feeding whitefly, *Bemisia tabaci*, represses plant jasmonic acid defenses against insects via salicylic acid-jasmonic acid antagonism with the help of its *Hamiltonella defensa* symbionts (Su et al. 2015). Oral secretions of *Spodoptera litura* containing *Staphylococcus epidermidis* were also shown to induce salicylic acid and repress jasmonic acid (Yamasaki et al. 2021) Likewise, the cabbage looper moth *Trichoplusia ni* benefits from *P. syringae*-induced salicylic acid–jasmonic acid antagonism (Groen et al. 2013). However, *P. syringae* bacteria producing the jasmonic acid-mimic coronatin, that promotes stomatal opening and allowing for easy passage of microbes into the plant’s apoplast, may be disadvantageous to generalist insects through repression of salicylic acid defenses. As shown by Van Oosten (2007), specialist insect herbivores are not effected by salicylic acid repression, most probably due to their extraordinary good adaptation to cope with host plant defenses. Besides the antagonism between salicylic acid and jasmonic acid, insects and their associated microbes may also directly repress plant jasmonic acid defenses. White fly *B. tabaci* is a vector of the tomato yellow leaf curl virus C2 protein that was found to repress plant jasmonic acid responses to insect feeding, while promoting insect survival and reproduction (Shi et al. 2014). The viral C2 protein was found to prevent degradation of plant JASMONATE ZIM DOMAIN (JAZ) transcriptional repressor proteins required for plant jasmonic acid responses (Li et al. 2019). Aphids are also known to vector many viruses that repress plant defenses, including the cucumber mosaic virus (CMV; Carr et al. 2020). The CMV 2b protein was shown to repress plant jasmonic acid responses and its absence during infection made plants strongly resistant to aphids, again showing the importance of insect-associated microbes in insect–plant interactions (Ziebell et al. 2011).

### Induced resistance

Plants have different enhanced states of defense responsiveness that influence both insects and their microbiota (Fig.   2). Plant roots (rhizosphere) and above ground (phyllosphere) microbiota, associated with beneficial growth promoting and defense enhancing soil-microbes, plays an important role in plant resistance to insects and pathogens (Pineda et al . 2017). For instance, plant beneficial rhizobacterium *K. oxytoca*, i.e. transmitted by insects, can induce systemic resistance (ISR), priming plants for jasmonic acid and ethylene defenses that protect against necrotrophic pathogens and insects (Park et al. 2009, Pieterse et al. 2014). This primed state allows the plant to respond more quickly and strongly to an encountered threat. An example of a pathogen that would be affected by ISR is *Pectobacterium*, a necrotrophic plant pathogen and gut inhabitant of cabbage root fly (*Delia radicum*) larvae (Van Den Bosch and Welte 2020, Van Den Bosch et al. 2020). Another type of an enhanced state of plant defenses is systemic acquired resistance (SAR). SAR is characterized by systemically increased levels of salicylic acid, a phenomenon in which a prior pathogen infection, that can be transmitted by an insect vector, triggeres pattern- or effector-triggered immunity, thereby priming uninfected systemic tissue to become more sensitive to salicylic acid-signaling against biotrophic and hemi-biotrophic pathogens. In addition, herbivore induced resistance refers to herbivore (DAMP or HAMP) induced systemic accumulation of proteinase inhibitors, which inhibits insect digestive enzymes (Pieterse et al. 2014).

### Soil-born legacy

A very exciting recent finding of how microbes and insects affect plant defenses is via a soil-born legacy (Fig. 2). After plant insect infestation or infection by (vector transmitted) pathogens, recruitment of a beneficial soil microbiota provides protective plant–soil feedback to plants grown on the same soil in following generations (Bakker et al. 2018, Friman et al. 2021, Hannula et al. 2021). In an elegant experiment by Friman et al. (2021), cabbage plants were treated with different herbivorous insects, *Plutella xylostella* caterpillars, *Brevicoryne brassicae* aphids or cabbage root fly *D. radicum* larvae. Subsequently the soil of these plants was used in a second generation of plants that were challenged with *D. radicum* larvae and insect performance was assessed. The results showed that the soil microbiota changed due to the insect that infested the cabbage plant. Furthermore, this microbial change resulted in a lower performance of *D. radicum* in the second generation of plants grown on the same soil.

### Plant volatiles

Plant defense signaling ultimately results in the production and emission of insect deterrent and parasitoid or vector attracting herbivore-induced plant volatiles (Fig. 2; Chauvin et al. 2013, Wenig et al. 2019). *Ca*. *L. asiaticus* and *Ca*. *Liberibacter psyllaurous* repress both salicylic acid and jasmonic acid defenses induced by their psyllid vector and lead to the emission of volatile methyl–salicylic acid, which attracts psyllid insects that vector the bacteria (Casteel et al. 2012, Mann et al. 2012). For *Phytoplasma*-infected apple trees, a similar scenario has been described. Infected trees emit a sesquiterpene, E-β-caryophyllene, that attracts *Cacopsylla picta* psyllids that act as vectors and are a pest of apple trees (Sugio et al. 2011). On the contrary, insect symbionts may also reduce plant volatile emission and with that prevent parasitoid attraction. Pea aphid (*A. pisum*) endosymbionts *H. defensa* reduced the systemic release of plant volatiles, decreasing parasitoid (*Aphidius ervi*) recruitment (Frago et al. 2017). Volatiles may also be produced by bacteria. Fruit flies (*Drosophila melanogaster*) are attracted to oviposit on fruit with volatile terpenes, like 2-methylisoborneol, produced by *Streptomyces* bacteria (Ho et al. 2020). However, larvae developing on *Streptomyces*-colonized fruit were subsequently killed off by the bacterium’s chemical arsenal, suggesting that this mechanism could be employed for biocontrol methods.

### Microbial detoxification of plant defensive compounds

Eventually, plant defense signaling results in the production of antifeeding compounds (e.g. lectins) and toxic secondary metabolites (e.g. phytoalexins, glycosides, and their breakdown products) that predominantly target the insects’ digestive system and affect the insects’ gut barrier (Miya et al. 2007, Schlaeppi et al. 2008, Mao et al. 2011, Van Den Borre et al. 2011, Bhargava et al. 2013, Kettles et al. 2013, Burow et al. 2015, Frerigmann et al. 2016, Wang et al. 2016, Hickman et al. 2017, Mason et al. 2019). Hundreds of secondary plant metabolites that affect insect herbivores are known, covering alkaloids, flavonoids, glucosinolates, terpenes, and fatty-acid-derived molecules (Fahey et al. 2001, D’Auria and Gershenzon 2005, Moco et al. 2006). Examples of well-known plant secondary metabolites with insecticidal properties are solanine, tomatine, caffeine, and nicotine. Although insects have a short food retention time, toxicity can be a major threat. Since insects have coevolved with their host plants, they harbor a wealth of adaptations that allow them to feed on plants with specialized defense mechanisms (Beran et al. 2019). These resistance mechanisms include target-site adaptations, inactivation via gut alkalization, rapid excretion, sequestration, degradation, and detoxification via cytochrome P450 monooxygenases, glutathione transferases, and carboxylesterases (Li et al. 2007, Winde and Wittstock 2011). Over 660 P450 monooxygenases are known in insects and together with their associated reductases these enzymes confer insect resistance to all known pesticide classes (Feyereisen 2005). In addition, feeding on toxic plants poses a major selective pressure on both the insect and their gut microbiota, suggesting that microbial adaptations that could benefit their host insect are likely to occur (Vilanova et al. 2016). Several authors reported the relevant role of symbiotic microorganisms in terms of detoxification and their impact on insect’s performance (Capuzzo et al. 2005, Kikuchi et al.2011b, Ceja-Navarro et al. 2015, Berasategui et al. 2017, Pavlidi et al. 2017, Comandatore et al. 2021). For instance, Ceja-Navarro et al. (2015) demonstrated that microbiota-free insects were no longer capable of caffeine and oleuropein degradation. Detoxifying microbial symbiosis is a unique strategy that insects adopt in coping with toxic secondary plant metabolites and insecticides and it is currently one of the most serious problems in agriculture (Van den Bosch and Welte 2017, Sato et al. 2021).

### Concluding remarks and future directions

In the interaction between insects and plants, insect-associated microbes play important roles in shaping plant responses (Atkinson and Urwin 2012, Coolen et al. 2016, Shikano et al. 2017). How microbes achieve this, remains largely unknown and gives rise to an exciting and rapidly expanding field of research. Many insects are associated with microbes, some of which are known to be phytopathogenic or able to influence plant defenses to the benefit of their host insect. Insect’s microbial diversity is enormous and we are only starting to understand their abilities in shaping insect–plant interactions. Microbes can counteract plant defenses on different levels, from preventing defense activation and manipulating plant phytohormonal signaling to detoxification of plant secondary metabolites. The insect’s microbiota was even shown to determine whether an insect is considered a pest because of microbial alterations that determine an insect’s plant host range.

Multitrophic interactions are distinct from single interactions, making them hard to predict and lead to unexpected outcomes (Atkinson et al. 2013, Coolen et al. 2016, Thoen et al. 2017). The same holds true for interactions between the insect’s microbiota and host plants. Furthermore, one should carefully consider the complexity of insect–plant interactions, even beyond microorganisms. Interplant communication via volatile alarm signals may prime neighboring plants for defenses against insects and pathogens that are perceived by other plants. Parental plants may even leave epigenetic signatures for their offspring in the form of heritable modifications that provide protection against threats in following generations (Boyko et al. 2010, Heil and Karban 2010, Bilichak et al. 2012, Dowen et al. 2012, Rasmann et al. 2012, Dicke 2016). Plants may also encounter multiple insects that alter interactions with subsequent insects and their associated microbes, and the presence of multiple microbes or pathogens can have different outcomes depending on the lifestyle of the pathogen and the plant’s response (Mitsuhashi et al. 2002, Schoonhoven et al. 2005, Bernays 2009, Rodriguez-Saona et al. 2010, Zhang et al. 2013, Stam et al. 2014, Krstić et al. 2018, Stork 2018).

Considering future perspectives, plants and crops are likely to encounter complex multitrophic interactions more frequently, as our changing climate with extreme weather conditions will support insects and pathogens to spread more easily and even beyond seasons where they normally occur (Chakraborty and Newton 2011, Bebber et al. 2013, Garrett et al. 2013). Together with our rapidly increasing human population, reaching 9.3 billion people by 2050, serious problems for food security are predicted (Mittler and Blumwald 2010, Newton et al. 2011, Teixeira et al. 2013). A dramatic increase in efficient food production is required in order to meet future food demands (UN 2011, FAO 2012). To reach these demands, pest management is of great importance. Where pesticides were once key tools for pest reduction, the usage of these products is greatly reduced nowadays, because of health risks and resistance buildup (Gilden et al. 2010, Meissle et al. 2010, Gressel 2011). Therefore, using and improving natural adaptive mechanisms of plants, insects and their associated microbes may provide sustainable alternatives without adversely affecting the ecological footprint. In order to effectively develop pest and plant disease management strategies, more knowledge on the multitrophic interactions between insects, plants and their associated microbes is required. Insect-associated microbes may be excellent targets for pest control, since insects rely on their microbial services.
