# Correction to: The secret life of insect-associated microbes and how they shape insect–plant interactions

**DOI:** 10.1093/femsec/fiac116

**Published:** 2022-10-10

This is a correction to: Silvia Coolen, Magda Rogowska-van der Molen, Cornelia U Welte, The secret life of insect-associated microbes and how they shape insect–plant interactions, FEMS Microbiology Ecology, Volume 98, Issue 9, September 2022, fiac083, https://doi.org/10.1093/femsec/fiac083

In the originally published version of this manuscript, the name of author Magda Rogowska-van der Molen was erroneously given as Rogowska-van der-Molen Magda.

This error has been corrected.

